# LA velocities and stasis assessed by 4D flow MRI are closely associated with LAA peak velocities by Doppler TEE in patients with atrial fibrillation

**DOI:** 10.1186/1532-429X-18-S1-P1

**Published:** 2016-01-27

**Authors:** Michael Mark, Nicholas Furiasse, Daniel C Lee, Jason Ng, James C Carr, Jeffrey J Goldberger

**Affiliations:** grid.465264.7Northwestern University, Chicago, IL USA

## Background

Atrial fibrillation (AF) is associated with increased risk of stroke due to development of left atrial (LA) thrombus [1]. Studies utilizing transesophageal echocardiography (TEE) have shown that decreased peak emptying flow velocity in the LA appendage (LAA) in patients with AF is a risk factor for stroke [2, 3]. As a non-invasive alternative, 4D flow MRI can measure 3D blood flow velocities with full coverage of the LA [4]. However, 4D flow data are acquired over multiple heart beats and beat-to-beat flow variations in AF patients with cardiac arrhythmia cannot be captured. A recent simulation study showed that ECG gated multi-beat flow MRI in patients with cardiac arrhythmia could reproduce persistent average LA and left ventricular mean velocities within 7.0-7.4% compared to real time TEE [5]. These findings suggest that 4D flow MRI can reliably assess persistent atrial flow and stasis patterns that are consistently present over multiple heart beats. However, a systematic comparison of LA flow dynamics based 4D flow MRI and real time TEE (reference standard) in-vivo is lacking. Our aim was thus to test the hypothesis that 4D flow MRI derived metrics of LA flow (velocities, stasis) are associated with LAA peak velocities obtained by TEE in the same patient.

## Methods

2D Doppler TEE and 4D flow MRI were performed in 30 AF patients (20 male, age = 62 ± 9 years, LVEF = 57 ± 7%). TEE based LAA emptying velocities were quantified. 4D flow MRI was employed to measure time-resolved 3D LA blood flow velocities. 3D segmentation of the LA was used to isolate LA velocities and to quantify LA flow parameters (mean, median, peak LA velocities) and stasis (% of LA exposed to velocities < 0.1 m/s).

## Results

**Figure 1:**
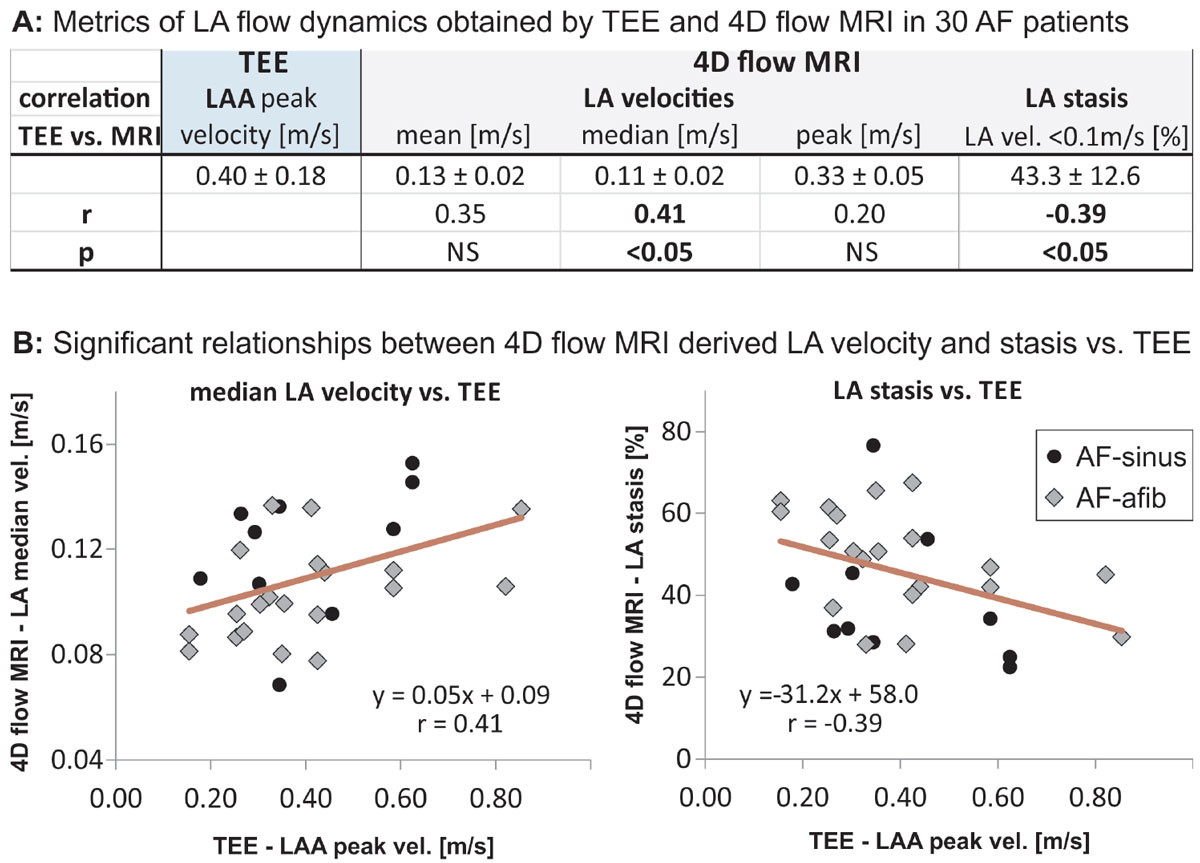
Figure 1

Time difference between TEE and 4D flow MRI was 41 ± 68 days, 10 patients were in sinus rhythm (AF-sinus), and 20 were in AF (AF-afib) during both TEE and MRI. As summarized in table and figure 1, Peak velocities in the entire LA measured by 4D flow MRI were significantly lower by 20% compared to LAA peak velocities in TEE (0.33 ± 0.05 m/s vs. 0.40 ± 0.18 m/s, p = 0.03). There were significant relationships (p < 0.05) between TEE LAA peak emptying velocities and 4D flow based LA median velocities (0.11 ± 0.02 m/s, r = 0.41) and LA stasis (45.5 ± 14.2%, r = -0.39).

## Conclusions

The findings of this study demonstrate that 4D flow MRI can characterize LA flow dynamics compared to the reference standard TEE, even in presence of arrhythmias during the MRI scan (present in 2/3 of patients in our study cohort). The inverse relationship between LAA peak velocities in TEE with LA stasis by 4D flow MRI indicates the potential of 4D flow MRI to assess impaired LA flow dynamics as a less invasive diagnostic alternative to TEE.
